# Supplementary material for: Hot spot prediction in protein-protein interactions by an ensemble system
Source: BMC Syst Biol. 2018 Dec 31;12(Suppl 9):132. doi: 10.1186/s12918-018-0665-8 (PMC6311905; doi:10.1186/s12918-018-0665-8)
Supplement: Supplementary file 2 — All relevant MATLAB code. A simple Matlab implement of our predictor. (ZIP 19,742 kb) [file 12918_2018_665_MOESM2_ESM.zip › Additional file 2/Read ASA/ASA website/dbMPIKT/1YCSA.html]

 NetSurfP - Protein Surface Accessibility and Secondary Structure Predictions

  

|  |  |  |
| --- | --- | --- |
|  |  | NetSurfP - Protein Surface Accessibility and Secondary Structure Predictions  Technical University of Denmark |

  


---

```
# For publication of results, please cite:
# A generic method for assignment of reliability scores applied to solvent accessibility predictions.
# Bent Petersen, Thomas Nordahl Petersen, Pernille Andersen, Morten Nielsen and Claus Lundegaard
# BMC Structural Biology 2009, 9:51 doi:10.1186/1472-6807-9-51
#
# Column 1: Class assignment - B for buried or E for Exposed - Threshold: 25% exposure, but not based on RSA
# Column 2: Amino acid
# Column 3: Sequence name
# Column 4: Amino acid number
# Column 5: Relative Surface Accessibility - RSA
# Column 6: Absolute Surface Accessibility
# Column 7: Z-fit score for RSA prediction
# Column 8: Probability for Alpha-Helix
# Column 9: Probability for Beta-strand
# Column 10: Probability for Coil
E S  1YCSA                  1    0.888 104.027  -0.056   0.003   0.003   0.994
E S  1YCSA                  2    0.445  52.119  -2.252   0.005   0.015   0.979
E S  1YCSA                  3    0.607  71.164  -1.525   0.018   0.047   0.935
E V  1YCSA                  4    0.346  53.196  -0.718   0.018   0.047   0.935
E P  1YCSA                  5    0.326  46.203  -1.022   0.018   0.019   0.964
E S  1YCSA                  6    0.427  49.998  -0.556   0.018   0.047   0.935
E Q  1YCSA                  7    0.487  87.032  -0.356   0.018   0.088   0.893
E K  1YCSA                  8    0.615 126.588  -0.484   0.018   0.088   0.893
E T  1YCSA                  9    0.663  91.972  -0.645   0.018   0.088   0.893
B Y  1YCSA                 10    0.159  33.871   0.908   0.018   0.047   0.935
E Q  1YCSA                 11    0.435  77.745  -0.685   0.018   0.047   0.935
E G  1YCSA                 12    0.304  23.948  -0.825   0.018   0.088   0.893
E S  1YCSA                 13    0.540  63.253  -0.569   0.005   0.262   0.733
B Y  1YCSA                 14    0.167  35.602   0.557   0.004   0.420   0.576
B G  1YCSA                 15    0.306  24.066  -0.861   0.004   0.514   0.481
B F  1YCSA                 16    0.051  10.316   0.792   0.002   0.816   0.182
E R  1YCSA                 17    0.361  82.783   1.398   0.001   0.900   0.099
B L  1YCSA                 18    0.052   9.503   0.998   0.001   0.900   0.099
E G  1YCSA                 19    0.384  30.213   1.133   0.001   0.900   0.099
B F  1YCSA                 20    0.106  21.234  -0.118   0.003   0.718   0.279
E L  1YCSA                 21    0.490  89.682   0.071   0.004   0.420   0.576
E H  1YCSA                 22    0.483  87.785  -0.157   0.004   0.197   0.799
E S  1YCSA                 23    0.440  51.591  -0.114   0.018   0.088   0.893
E G  1YCSA                 24    0.725  57.073  -1.223   0.018   0.019   0.964
E T  1YCSA                 25    0.474  65.730  -0.781   0.018   0.019   0.964
E A  1YCSA                 26    0.483  53.183  -1.908   0.018   0.047   0.935
E K  1YCSA                 27    0.599 123.255  -0.716   0.018   0.047   0.935
E S  1YCSA                 28    0.429  50.326  -1.432   0.018   0.047   0.935
E V  1YCSA                 29    0.388  59.651  -0.543   0.018   0.088   0.893
E T  1YCSA                 30    0.348  48.268  -0.892   0.064   0.216   0.721
B C  1YCSA                 31    0.151  21.228   0.550   0.074   0.484   0.442
B T  1YCSA                 32    0.293  40.584  -0.432   0.023   0.655   0.322
B Y  1YCSA                 33    0.172  36.863   0.087   0.074   0.484   0.442
B S  1YCSA                 34    0.211  24.729   0.380   0.502   0.102   0.396
E P  1YCSA                 35    0.608  86.218   0.198   0.717   0.014   0.269
E A  1YCSA                 36    0.385  42.471   0.956   0.717   0.014   0.269
B L  1YCSA                 37    0.055  10.071   0.546   0.622   0.015   0.363
E N  1YCSA                 38    0.403  58.970   0.725   0.622   0.015   0.363
E K  1YCSA                 39    0.392  80.676   1.189   0.605   0.105   0.290
B M  1YCSA                 40    0.078  15.688   0.504   0.478   0.309   0.214
B F  1YCSA                 41    0.127  25.409   0.592   0.268   0.505   0.227
B C  1YCSA                 42    0.089  12.538   0.461   0.169   0.612   0.219
E Q  1YCSA                 43    0.407  72.762   0.796   0.074   0.484   0.442
B L  1YCSA                 44    0.184  33.654   0.006   0.019   0.141   0.840
E A  1YCSA                 45    0.651  71.762  -0.331   0.018   0.047   0.935
E K  1YCSA                 46    0.372  76.500   0.953   0.018   0.088   0.893
E T  1YCSA                 47    0.440  60.986   0.585   0.005   0.262   0.733
B C  1YCSA                 48    0.050   7.048   1.333   0.004   0.420   0.576
E P  1YCSA                 49    0.239  33.971   0.650   0.003   0.718   0.279
B V  1YCSA                 50    0.054   8.361   0.656   0.001   0.900   0.099
E Q  1YCSA                 51    0.370  66.011   1.357   0.001   0.959   0.040
B L  1YCSA                 52    0.047   8.551   0.368   0.001   0.959   0.040
E W  1YCSA                 53    0.305  73.304   0.753   0.001   0.959   0.040
B V  1YCSA                 54    0.129  19.904   0.099   0.001   0.900   0.099
E D  1YCSA                 55    0.544  78.448  -0.099   0.004   0.616   0.381
E S  1YCSA                 56    0.502  58.893  -0.994   0.004   0.197   0.799
E T  1YCSA                 57    0.530  73.553  -0.393   0.005   0.045   0.951
E P  1YCSA                 58    0.264  37.419   0.032   0.005   0.015   0.979
E P  1YCSA                 59    0.264  37.419  -0.643   0.003   0.003   0.994
E P  1YCSA                 60    0.707 100.394  -0.081   0.018   0.019   0.964
E G  1YCSA                 61    0.511  40.176  -0.531   0.005   0.015   0.979
B T  1YCSA                 62    0.045   6.214   0.227   0.004   0.138   0.858
B R  1YCSA                 63    0.189  43.189   0.592   0.002   0.816   0.182
B V  1YCSA                 64    0.022   3.397   1.279   0.001   0.959   0.040
B R  1YCSA                 65    0.085  19.373   0.818   0.001   0.959   0.040
B A  1YCSA                 66    0.023   2.535   0.439   0.001   0.959   0.040
B M  1YCSA                 67    0.061  12.166   1.067   0.001   0.959   0.040
B A  1YCSA                 68    0.029   3.229   0.560   0.001   0.959   0.040
B I  1YCSA                 69    0.041   7.567  -0.011   0.011   0.918   0.071
B Y  1YCSA                 70    0.052  11.134  -0.166   0.003   0.718   0.279
E K  1YCSA                 71    0.269  55.436   0.188   0.005   0.336   0.660
E Q  1YCSA                 72    0.372  66.511  -0.099   0.005   0.045   0.951
E S  1YCSA                 73    0.323  37.856  -0.648   0.018   0.047   0.935
E Q  1YCSA                 74    0.600 107.214  -0.919   0.113   0.043   0.844
E H  1YCSA                 75    0.298  54.279  -0.292   0.113   0.043   0.844
B M  1YCSA                 76    0.178  35.698  -0.112   0.278   0.093   0.628
E T  1YCSA                 77    0.406  56.340   0.205   0.216   0.235   0.548
E E  1YCSA                 78    0.386  67.364   0.842   0.321   0.252   0.427
B V  1YCSA                 79    0.175  26.836   0.858   0.453   0.248   0.299
B V  1YCSA                 80    0.090  13.787  -0.333   0.352   0.332   0.316
E R  1YCSA                 81    0.472 108.202   1.339   0.352   0.332   0.316
E R  1YCSA                 82    0.309  70.830   1.058   0.321   0.252   0.427
B C  1YCSA                 83    0.036   5.040   0.771   0.113   0.087   0.800
E P  1YCSA                 84    0.495  70.212   0.156   0.113   0.043   0.844
E H  1YCSA                 85    0.435  79.090  -0.168   0.053   0.043   0.903
B H  1YCSA                 86    0.178  32.360  -0.282   0.113   0.087   0.800
E E  1YCSA                 87    0.422  73.688  -0.164   0.118   0.150   0.732
E R  1YCSA                 88    0.453 103.783   0.092   0.052   0.084   0.864
E C  1YCSA                 89    0.373  52.369  -0.600   0.053   0.043   0.903
E S  1YCSA                 90    0.693  81.173  -1.174   0.018   0.019   0.964
E D  1YCSA                 91    0.652  93.996  -0.974   0.018   0.019   0.964
E S  1YCSA                 92    0.487  57.100  -2.032   0.018   0.019   0.964
E D  1YCSA                 93    0.670  96.576  -0.889   0.018   0.019   0.964
E G  1YCSA                 94    0.553  43.560  -1.836   0.016   0.005   0.979
E L  1YCSA                 95    0.521  95.358  -1.715   0.018   0.019   0.964
E A  1YCSA                 96    0.469  51.695  -0.368   0.016   0.005   0.979
E P  1YCSA                 97    0.293  41.548   0.047   0.016   0.005   0.979
E P  1YCSA                 98    0.328  46.586   0.120   0.018   0.019   0.964
E Q  1YCSA                 99    0.396  70.690   0.969   0.018   0.088   0.893
B H  1YCSA                100    0.063  11.387   0.233   0.004   0.420   0.576
B L  1YCSA                101    0.034   6.317  -0.202   0.002   0.816   0.182
B I  1YCSA                102    0.029   5.365   0.560   0.001   0.900   0.099
B R  1YCSA                103    0.108  24.618   0.465   0.001   0.900   0.099
B V  1YCSA                104    0.040   6.133   0.191   0.002   0.816   0.182
E E  1YCSA                105    0.385  67.312   0.216   0.004   0.420   0.576
E G  1YCSA                106    0.476  37.477  -1.467   0.004   0.138   0.858
E N  1YCSA                107    0.451  65.982  -0.210   0.004   0.085   0.910
E L  1YCSA                108    0.462  84.556  -1.126   0.004   0.085   0.910
E R  1YCSA                109    0.433  99.271   0.676   0.020   0.205   0.775
B V  1YCSA                110    0.059   9.145  -0.791   0.021   0.756   0.223
E E  1YCSA                111    0.338  58.979   1.272   0.011   0.918   0.071
B Y  1YCSA                112    0.173  36.949  -0.146   0.011   0.918   0.071
E L  1YCSA                113    0.280  51.250   0.367   0.021   0.756   0.223
E D  1YCSA                114    0.379  54.571  -1.113   0.004   0.420   0.576
B D  1YCSA                115    0.265  38.259  -0.151   0.004   0.085   0.910
E R  1YCSA                116    0.713 163.369  -0.287   0.018   0.047   0.935
E N  1YCSA                117    0.560  81.984  -0.632   0.018   0.047   0.935
E T  1YCSA                118    0.277  38.475  -0.523   0.019   0.141   0.840
E F  1YCSA                119    0.294  59.026   0.328   0.005   0.336   0.660
B R  1YCSA                120    0.189  43.373   0.656   0.003   0.718   0.279
B H  1YCSA                121    0.075  13.715   0.814   0.001   0.900   0.099
B S  1YCSA                122    0.054   6.364  -0.512   0.001   0.959   0.040
B V  1YCSA                123    0.024   3.750   0.596   0.001   0.959   0.040
B V  1YCSA                124    0.091  13.910   0.287   0.001   0.959   0.040
B V  1YCSA                125    0.040   6.102   0.307   0.003   0.718   0.279
B P  1YCSA                126    0.088  12.501  -1.534   0.005   0.336   0.660
B Y  1YCSA                127    0.181  38.765  -1.088   0.004   0.138   0.858
E E  1YCSA                128    0.487  85.114  -1.063   0.004   0.138   0.858
E P  1YCSA                129    0.344  48.814  -0.131   0.005   0.045   0.951
B P  1YCSA                130    0.304  43.166  -0.845   0.018   0.047   0.935
E E  1YCSA                131    0.518  90.495  -0.977   0.018   0.088   0.893
E V  1YCSA                132    0.352  54.072  -1.740   0.018   0.088   0.893
E G  1YCSA                133    0.539  42.411  -1.233   0.018   0.088   0.893
E S  1YCSA                134    0.374  43.880  -0.718   0.022   0.359   0.619
E D  1YCSA                135    0.463  66.776  -0.243   0.021   0.756   0.223
E C  1YCSA                136    0.339  47.596   0.236   0.011   0.918   0.071
B T  1YCSA                137    0.204  28.253   0.724   0.011   0.918   0.071
B T  1YCSA                138    0.292  40.500   0.096   0.018   0.846   0.136
B I  1YCSA                139    0.135  24.956   0.680   0.018   0.846   0.136
B H  1YCSA                140    0.146  26.576  -0.083   0.085   0.775   0.140
B Y  1YCSA                141    0.055  11.753   0.336   0.085   0.775   0.140
B N  1YCSA                142    0.235  34.404   0.564   0.072   0.855   0.073
B Y  1YCSA                143    0.050  10.706   0.484   0.072   0.855   0.073
B M  1YCSA                144    0.098  19.610   0.888   0.072   0.855   0.073
B C  1YCSA                145    0.019   2.597   0.842   0.087   0.683   0.230
B N  1YCSA                146    0.198  28.958  -0.659   0.066   0.296   0.638
B S  1YCSA                147    0.176  20.604  -2.727   0.056   0.142   0.802
B S  1YCSA                148    0.171  20.018  -1.046   0.056   0.142   0.802
B C  1YCSA                149    0.065   9.126  -1.363   0.052   0.084   0.864
E M  1YCSA                150    0.376  75.138  -0.572   0.053   0.043   0.903
E G  1YCSA                151    0.412  32.464  -2.266   0.053   0.043   0.903
E G  1YCSA                152    0.414  32.598  -1.039   0.113   0.043   0.844
B M  1YCSA                153    0.230  45.983  -1.601   0.113   0.043   0.844
E N  1YCSA                154    0.445  65.148  -0.894   0.113   0.043   0.844
E R  1YCSA                155    0.299  68.517   0.071   0.113   0.087   0.800
B R  1YCSA                156    0.219  50.059   0.468   0.125   0.227   0.648
B P  1YCSA                157    0.189  26.791   0.411   0.074   0.484   0.442
B I  1YCSA                158    0.043   8.011  -0.175   0.021   0.756   0.223
B L  1YCSA                159    0.058  10.656   0.310   0.011   0.918   0.071
B T  1YCSA                160    0.026   3.606   0.525   0.006   0.962   0.032
B I  1YCSA                161    0.022   3.996   0.970   0.001   0.959   0.040
B I  1YCSA                162    0.023   4.200   1.203   0.001   0.959   0.040
B T  1YCSA                163    0.056   7.809   0.882   0.001   0.959   0.040
B L  1YCSA                164    0.032   5.804   0.424   0.001   0.900   0.099
B E  1YCSA                165    0.224  39.220   0.109   0.002   0.816   0.182
B D  1YCSA                166    0.237  34.195   0.291   0.005   0.336   0.660
E S  1YCSA                167    0.621  72.816  -0.979   0.005   0.045   0.951
E S  1YCSA                168    0.725  84.982  -0.283   0.005   0.015   0.979
E G  1YCSA                169    0.355  27.962  -1.031   0.005   0.045   0.951
E N  1YCSA                170    0.430  62.908   0.291   0.004   0.420   0.576
B L  1YCSA                171    0.091  16.735   0.040   0.002   0.816   0.182
B L  1YCSA                172    0.145  26.568   0.602   0.002   0.816   0.182
B G  1YCSA                173    0.085   6.666  -0.721   0.003   0.718   0.279
B R  1YCSA                174    0.267  61.097   0.536   0.002   0.816   0.182
E N  1YCSA                175    0.320  46.789  -0.134   0.002   0.816   0.182
B S  1YCSA                176    0.141  16.537   0.149   0.001   0.900   0.099
B F  1YCSA                177    0.077  15.474   0.460   0.001   0.900   0.099
E E  1YCSA                178    0.331  57.773   0.570   0.001   0.900   0.099
B V  1YCSA                179    0.033   5.087   0.471   0.001   0.900   0.099
E R  1YCSA                180    0.265  60.616   0.710   0.001   0.900   0.099
B V  1YCSA                181    0.053   8.161   0.709   0.018   0.846   0.136
B C  1YCSA                182    0.040   5.574  -0.239   0.023   0.655   0.322
E A  1YCSA                183    0.293  32.300  -0.378   0.021   0.451   0.528
B C  1YCSA                184    0.153  21.495   0.208   0.018   0.088   0.893
E P  1YCSA                185    0.299  42.386  -0.099   0.018   0.047   0.935
E G  1YCSA                186    0.507  39.901  -0.155   0.058   0.017   0.925
E R  1YCSA                187    0.415  94.943   0.041   0.181   0.016   0.803
E D  1YCSA                188    0.441  63.563  -0.338   0.181   0.016   0.803
E R  1YCSA                189    0.368  84.364   0.388   0.622   0.015   0.363
E R  1YCSA                190    0.609 139.530   0.920   0.782   0.003   0.216
E T  1YCSA                191    0.539  74.745   0.993   0.858   0.002   0.139
E E  1YCSA                192    0.425  74.335  -0.005   0.923   0.002   0.076
E E  1YCSA                193    0.494  86.214  -0.044   0.923   0.002   0.076
E E  1YCSA                194    0.622 108.733   0.872   0.858   0.002   0.139
E N  1YCSA                195    0.473  69.306   0.005   0.782   0.003   0.216
E L  1YCSA                196    0.426  77.964  -0.295   0.694   0.003   0.303
E R  1YCSA                197    0.680 155.720   0.289   0.600   0.003   0.397
E K  1YCSA                198    0.674 138.704   0.367   0.406   0.004   0.590
E K  1YCSA                199    0.843 173.364   0.896   0.003   0.003   0.994

Explain the output.  Go back.
```

---
